# Ataxia in children: think about vitamin E deficiency ! (comment on: ataxia in children: early recognition and clinical evaluation)

**DOI:** 10.1186/s13052-017-0378-4

**Published:** 2017-07-19

**Authors:** H. Rahmoune, N. Boutrid, M. Amrane, M. C. Chekkour, B. Bioud

**Affiliations:** 1 Pediatrics Department, Setif University Hospital, Genetic & Nutritional Diseases Laboratory, Setif-1 University, 19000 Setif, Algeria; 2 Genetic & Nutritional Diseases Laboratory, Setif-1 University, 19000 Setif, Algeria; 3 Biochemistry Department, Setif University Hospital, 19000 Setif, Algeria; 4 Neurology Department, Setif University Hospital, 19000 Setif, Algeria

**Keywords:** Ataxia, Vitamin E deficiency, Genetic, Mediterranean

## Abstract

A recent article from Pavone et al. published in the Italian Journal of Pediatrics entitled «Ataxia in children: early recognition and clinical evaluation» made an exhaustive overview of the large spectrum of pediatric ataxias.

However, we would underline the importance of considering hereditary ataxia due to isolated vitamin E deficiency as a specific and treatable cause of child ataxia.

We present a short clinical and therpeutic synopsis of this peculiar genetic etiology, frequently encountred in the mediterranean region.

## Background

A recent review published in The Italian Journal of Paediatrics focused on pediatric ataxia and its different causes, acquired and genetic [1].

It should be important to specify that among the myriad of herediatary ataxias, only few autosomal recessive types have dietary or biochemical therapies: ataxia with vitamin E deficiency, cerebrotendinous xanthomatosis, Refsum Disease, abetalipoproteinemia, Niemann Pick C disease and coenzyme Q10 deficiency [2].

The ataxia with vitamin E deficiency (AVED) is a peculiar one and deserves a special attention.

## Main text

AVED generally manifests in late childhood or early teens with dysarthria, poor balance when walking (especially in the dark), and progressive clumsiness.

Phenotypically similar to Friedreich ataxia, AVED is more frequently associated with head titubation or dystonia, and less with cardiomyopathy [2].

This clincal phenotype constitutes a large spectrum: severity may vary among sibs (personal data) while the course tend to be uniform within the same family [3].

AVED is caused by mutations in the tocopherol (alpha) transfer protein gene (TTPA; 8q13).

This protein binds alpha-tocopherol (a vitamin E isomer) and very-low-density lipoproteins (VLDLs) in the liver. When mutated, TTPA prevents vitamin E linking to VLDLs, preventing it to pass into general circulation. [4]. The majority of the reported cases originated in the Mediterranean region, and the 744delA mutation (more specific in early onset form) was the most common among 22 known mutants [5].

Nevertheless, all forms of pediatric ataxia everywhere should be tested for AVED to make an early diagnosis, initiate treatment with high dose vitamin E, and avoid subsequent severe neurological impairment [6]. AVED can be easily confirmed with a blood sample (very low vitamin E, normal lipids and liporproteins profiles), along with a definitive exclusion of Friedreich ataxia, the closest form of such constitutional disorder [7].

During vitamin E supplementation, the plasma vitamin E concentration should be measured at regular intervals (e.g., every 6 months) in order to maintain it in the high normal range [8].

At the opposite, acquired vitamin E deficiency is most be observed in pediatric chronic diseases impeding ileal resorption of fat-soluble vitamins; e.g. cystic fibrosis, Crohn’s disease, cholestatic liver disease or short bowel syndrome [9]. Even if symptoms seem similar to AVED, preventive or curative supplementation with oral vitamin E requires less doses than the huge posology necessary to treat AVED (800–1500 mg/day) [10].

## Conclusion

Diagnosis of AVED (easily made by first measuring serum concentration of vitamin E) needs to be considered in every suspected genetic ataxia.

This particular ataxia, frequent in the Mediterranean area, offers a dramatic potential of both prevention (i.e. intrafamilial screening) and cure.

## Abbreviations

AVED: Ataxia with vitamin E deficiency
VLDLs: Very-low-density lipoproteins

## References

[1] Pavone P, Andrea D, Praticò AD, Pavone V, Lubrano R, Falsaperla R, Rizzo R, Ruggieri M (2017). Ataxia in children: early recognition and clinical evaluation. Ital J Pediatr.

[2] Jayadev S, Bird TD (2013). Hereditary ataxias: overview. Genet Med.

[3] Shorer Z, Parvari R, Bril G, Sela BA, Moses S (1996). Ataxia with isolated vitamin E deficiency in four siblings. Pediatr Neurol.

[4] Anheim M. Ataxia with vitamin E deficiency. 2013. Orphanet Database. http://www.orpha.net/consor/cgi-bin/OC_Exp.php?lng=EN&Expert=96. Accessed 16 Jan 2017.

[5] El Euch-Fayache G, Bouhlal Y, Amouri R, Feki M, Hentati F (2014). Molecular, clinical and peripheral neuropathy study of Tunisian patients with ataxia with vitamin E deficiency. Brain.

[6] Elkamil A, Johansen KK, Aasly J (2015). Ataxia with vitamin E deficiency in Norway. J Mov Disord.

[7] Bird TB. Hereditary Ataxia Overview.2016. In: GeneReviews®. https://www.ncbi.nlm.nih.gov/books/NBK1138/. Accessed 16 Jan 2017.

[8] Schuelke MD. Ataxia with Vitamin E Deficiency.2016. In: GeneReviews®. https://www.ncbi.nlm.nih.gov/books/NBK1241/. Accessed 16 Jan 2017.

[9] Göransson G, Nord'en A, Akesson B (1973). Low plasma tocopherol levels in patients with gastrointestinal disorders. Scand J Gastroenterol.

[10] Mariotti C, Gellera C, Rimoldi M, Mineri R, Uziel G, Zorzi G, Pareyson D, Piccolo G, Gambi D, Piacentini S, Squitieri F, Capra R, Castellotti B, Di Donato S (2004). Ataxia with isolated vitamin E deficiency: neurological phenotype, clinical followup and novel mutations in TTPA gene in Italian families. Neurol Sci.

